# Positive association between patient experience of primary care with chronic disease and self‐rated health in a Japanese rural area: A cross‐sectional questionnaire survey using the Japanese primary care assessment tool short form

**DOI:** 10.1002/jgf2.721

**Published:** 2024-07-29

**Authors:** Sakiya Nishida, Yoshio Hisata, Aki Yasaka, Takashi Sugioka, Risa Hirata, Naoko E. Katsuki, Masaki Tago, Yuki Ueda, Masaki Amenomori, Katsumi Higashino, Yoshio Naya

**Affiliations:** ^1^ Department of Internal Medicine Nagahama City Kohoku Hospital Nagahama Japan; ^2^ Department of Family Medicine Shiga Center for Family Medicine Gamo‐gun Japan; ^3^ Department of General Medicine Saga University Hospital Saga City Japan; ^4^ Department of Neurology National Hospital Organization Sendai Medical Center Sendai Japan; ^5^ Department of General Medicine Saga City Fuji‐Yamato Spa. Hospital Saga City Japan; ^6^ Department of Internal Medicine Nishiazai Clinic Nagahama Japan; ^7^ Department of Pediatrics Nagahama City Kohoku Hospital Nagahama Japan; ^8^ Department of Urology Nagahama City Kohoku Hospital Nagahama Japan

**Keywords:** Japanese primary care assessment tool short form (JPCAT‐SF), patient experience, primary care, rural area, self‐rated health (SRH)

## Abstract

**Background:**

Low birthrates and an aging society during population decline have brought about labor shortages and increased medical care needs. Medical resources should be distributed appropriately, particularly in rural areas. High‐quality primary care can make the medical system more effective and contribute to rural residents' health. However, there are no reports on the impact of primary care on health in the rural areas of Japan. This study examined the effect of primary care quality on self‐rated health in a rural Japanese area.

**Methods:**

We conducted a cross‐sectional study using a questionnaire survey of residents of a rural area in northern Shiga Prefecture in 2021. Self‐rated health (SRH) was used as the health outcome (1–5 points), and the Japanese Primary Care Assessment Tool Short Form (JPCAT‐SF) was used to measure the patients' experience of primary care (0–100 points). We conducted a multivariate analysis using logistic regression analysis and examined the association between good SRH (4 points or more) and high JPCAT‐SF scores (above average score), adjusted for sociodemographic confounding factors.

**Results:**

A total of 1108 men and 1412 women responded to the survey. The mean patient age was 69.8 years. The mean JPCAT‐SF score was 51.8 points. Among the 1172 patients with chronic diseases, good SRH was associated with high JPCAT‐SF scores (odds ratio [OR] 1.32, 95% confidence interval [CI] 1.009–1.73).

**Conclusions:**

Good SRH among rural residents with chronic disease was associated with high primary care quality.

## Abstract 2

This study examined the effect of primary care quality. Self‐rated health was used as the health outcome, and the Japanese Primary Care Assessment Tool Short Form (JPCAT‐SF) was used to measure the patients' experience of primary care. Good SRH was associated with high JPCAT‐SF scores.

## 1 INTRODUCTION

With Japan's declining birthrate and aging population, there is a need for reforms regarding the future management of healthcare and an appropriate understanding of healthcare needs.^1^ In 2022, 29.1% of the population is aged 65 years and over, and it is predicted to increase to 36.3% in 2045.^2^ Moreover, it is important to provide efficient healthcare access to ensure the health of this growing demographic.

Primary care is a multidimensional system that contributes to the overall healthcare delivery system and health.^3^ In rural areas, where the effective provision of healthcare resources is required, primary care functions could be essential to the health of the local population. Regarding the difference of primary care physicians in rural or urban areas in Japan, primary care physicians tended to have a broader scope of practice in rural areas than in urban areas.^4^ A wide range of medical treatments can lower hospitalization rates, reduce medical costs, and provide the appropriate care on time and proper place.^5^ Hence, the medical care provided by primary care physicians in rural areas could have a positive impact on the health of residents.

One of the many assessing instruments^6^ that measured primary care function was the Primary Care Assessment Tool (PCAT), and the Self‐Rated Health (SRH) predicted mortality^7^ and living conditions as simple health outcomes.^8^ Both PCAT and SRH are easily measurable and minimally invasive rating scales.

Globally, there are reports evaluating PCAT and SRH in Korea^9^ and China^10^; for example, in Korea, KPCT is used, and in China, PCAT‐T is used as an evaluation scale, and both scores were observed to be correlated with SRH score; however, in Japan, although there are reports examining differences in PCAT between hospitals and clinics,^11^ there are no reports examining the relationship between SRH in rural areas that are facing declining birth rates and aging population. We hypothesized that high‐quality primary care would positively influence people's health in Japanese rural areas.

This study aimed to investigate the health effects of the primary care provided by residents in rural Japanese areas. In this study, we discussed the characteristics of Japanese primary care in terms of medical treatment behaviors. The Japanese medical system adopts universal health insurance and free access; therefore, residents can always receive relatively inexpensive standard medical care.^1^ The residents without chronic diseases often visit medical institutions for the first visit and are directly seen by a specialist without referral or permission from their primary care physician. As such, the medical treatment behavior differs depending on whether patients have a disease or not. In addition, the first‐visit rate to clinics in Japan is approximately 13.4%^12^; therefore, the majority of patients visit clinics regularly for chronic diseases. As the latter group was expected to have many opportunities to be exposed to primary care, we decided to analyze them by classifying them according to the presence or absence of a chronic disease.

## 2 METHODS

### 2.1 Participants and period

The survey was conducted among residents of Kinomoto‐cho, Yogo‐cho, and Nishiazai‐cho in Nagahama City, Shiga Prefecture (Appendix S1). The above areas were designated as heavy snowfall and depopulated areas.^13^ Nagahama City Kohoku Hospital and Nishiazai Clinic are located in this area. The former was a public hospital, and the latter was managed by the Japan Association for the Development of Community Medicine. The Japan Association for the Development of Community Medicine is a public interest incorporated association that operates hospitals, clinics, and medical, health, welfare, and nursing care facilities under contract with local governments that have difficulty securing local medical care, including in rural areas.

As of April 2022, there were 6469 people living in Kinomoto, 2833 people in Yogo, and 3655 people in Nishiazai, with aging rates of 38.0%, 43.7%, and 38.7% that order.^14^ Of these, this survey was conducted among those aged 20 years and older.

In cases where respondents were unable to answer the questionnaire due to their nursing care needs or disabilities, family members living with them or their caregivers answered the questionnaire. Questionnaires were distributed and collected between April 2021 and April 2023. The reason it took 2 years to distribute and collect the questionnaires was due to the use of research funds and the amount of manpower required. Using two research grants, we distributed and collected the materials in two parts. Moreover, owing to the effects of the coronavirus pandemic, only a small group of people was assigned to create the mail and reply envelopes. Hence, a longer deadline was set to increase the collection rate.

The data for the three towns surveyed were named the KYANS STUDY, based on the initials of Kinomoto, Yogo, and Nishiazai‐cho in Shiga. KYANS also apply them to the similar word “kiyansu (‘きやんす’ in Japanese),” a Japanese dialect word meaning “people coming, including a nuance of respect” in the Kohoku (meaning north of Lake Biwa in Japanese) region. This study is an unofficial study and aimed to create a community in which people can gather and participate.

### 2.2 Research design

This observational cross‐sectional study used a questionnaire. That was distributed and collected by mail. Resident address information was provided by the City Hall. Questionnaires were mailed to all residents whose records were confirmed (exhaustive survey). This study complied with the STROBE guidelines.

### 2.3 Measurement items and definition of sociodemographic characteristics

The following participant attributes were examined: sex, age, educational background, marital status, employment status, and annual household income. The presence or absence of physical or mental diseases or disability was examined.

Male sex was used as the categorical variable. Age was categorized as “non‐elderly” if the age was less than 65 years old. Chronic diseases were defined as having any physical or mental disease or disability; those with any of these were defined as having a disease, and those with neither physical nor mental illness were defined as having no disease. Educational background was used in the analysis as “high educational background” if the respondent had a junior college or university degree or higher. Marital status was used in the analysis of “whether the respondent is married or not” as a confounding factor. Working status was defined in the analysis as “not working” if the respondent was unemployed or retired. Respondents were asked to answer on a 4‐point scale their annual household income (1.5, 3, and 5 million yen), and whether the income was 3.01 million yen or more in the middle was used for the analysis.

### 2.4 Definition of health status

Self‐rated health^7^, ^8^ was rated on a 5‐point scale: 5, very healthy; 4, fairly healthy; 3, intermediate; 2, not so healthy; and 1, not healthy at all. Among them, “very healthy” and “fairly healthy” were defined as “good health” and used for the analysis.

### 2.5 JPCAT‐SF as the main factor

The JPCAT‐SF proposed by Aoki et al. was used as a measure of primary care functions.^15^ The JPCAT is the first primary care quality assessment scale developed in Japan and consists of six subscales: first contact, continuity, coordination, comprehensiveness (services available), comprehensiveness (services provided), and community orientation. The JPCAT‐SF is a shortened version with fewer items than the JPCAT and was designed to reduce the burden on respondents. The validity and reliability of the JPCAT‐SF were completed.^16^ An in‐depth explanation of the JPCAT is provided in Appendix S2.

### 2.6 Statistical analysis

First, we described the measured items and results of the JPCAT‐SF. Next, we conducted a univariate analysis of the association between good SRH, high JPCAT‐SF scores, and sociodemographic characteristics using the chi‐square test. Finally, participants were divided into a whole group and two subgroups: (1) those who had a disease and a family doctor (subgroup with chronic disease), and (2) those not having a disease or family doctor (subgroup without chronic disease). Good SRH was used as the objective variable and high JPCAT‐SF scores were used as explanatory variables. Items that were significant in the univariate analysis were included as confounding factors, and multivariate logistic regression analysis was conducted for each group, adjusted for sex, age, education, marital status, employment, and income. STATA/BE (ver. 17.0) was used for analysis. All significance levels were set at *p* = 0.05. Outliers were excluded from the analysis if they were clearly misstated or incorrectly entered. Missing important outcomes or factor variables were excluded from analyses.

### 2.7 Ethical considerations

We distributed pre‐numbered questionnaires to each household by mail so that we could conduct additional surveys in the future and asked them to respond anonymously to the questionnaire. Care was taken not to identify individuals in the envelopes used to collect the questionnaires or data entrants. We obtained consent after clearly stating that, at the time of distribution, respondents were free to respond or withdraw their responses. This study was approved by the Ethics Committee of Nagahama City Kohoku Hospital (Approval number 2020 No. 3, 2021 No. 1).

## 3 RESULTS

**FIGURE 1:**
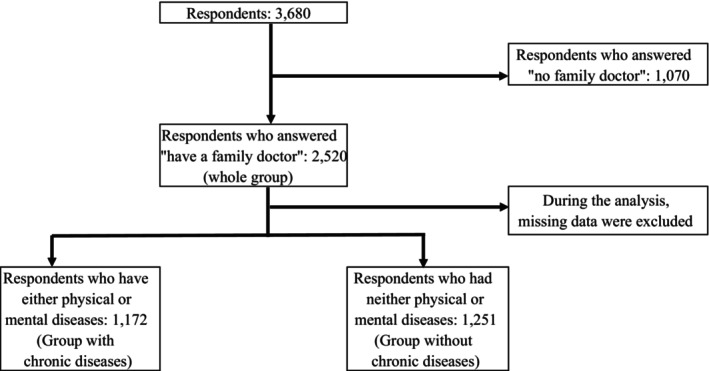
Inclusion criteria.

We administered the questionnaire to 11,381 people and obtained responses from 3680 people. The response rate was 32.3%. The 1070 respondents who answered “no family doctor” were excluded because they were not included in the JPCAT‐SF. When missing data were excluded, the number of respondents was 2520. The number of respondents who reported having a disease was 1172, and that of those who reported not having a disease was 1251 during the analysis (Figure 1).

### 3.1 Respondent attributes (Table 1)

**TABLE 1:** Respondent attributes, *N* = 2520.

	Number of cases (mean ± standard deviation)	Percentage (%)
*Sex*		
Male	1108	43.9
Female	1412	56.1
*Age*	69.8 ± 14.4	
Min	20	
Max	103	
<65 years old	719	
*Presence or absence of disease*		
Physical disease	1140	47
Mental disease	197	8.1
as either physical or mental disease	1172	48.3
No physical or mental disease	1251	51.6
*Self‐rated health* (*SRH*)		
Very healthy (①)	581	23.4
	(disease: 80, no disease: 501)	
Fairly healthy (②)	926	37.3
	(disease: 369, no disease: 557)	
Intermediate	523	21.1
	(disease: 335, no disease: 188)	
Not so healthy	341	13.8
	(disease: 298, no disease: 43)	
Not healthy at all	107	4.3
	(disease: 103, no disease: 4)	
Healthy group (① + ②)	1507	60.8
*Mean SRH score* (*1–5*)^a^	3.61 ± 1.11	
Mean SRH score with chronic disease	3.02 ± 1.08	
Mean SRH score without chronic disease	4.18 ± 0.80	
*Educational background*		
Middle school graduate	781	32
High school graduate	920	37.7
Junior college or university degree or higher	742	30.3
*Marital status*		
Unmarried	184	7.7
Married	1683	70.4
Divorced	54	2.3
Bereavement	469	19.6
*Employment status*		
In employment	903	36.7
Housewife/househusband	355	14.4
Student	5	0.2
Unemployed or retired	1198	48.7
*Annual household income*		
0–1.5 million yen	405	17.9
1.51–3 million yen	819	36.1
3.01–5 million yen	524	23.1
5.01 million yen or more	518	22.9

^a^ The mean SRH score was calculated using 1 point for not healthy at all, 2 points for not so healthy, 3 points for intermediate, 4 points for fairly healthy, and 5 points for very healthy.

This study included 1108 males and 1412 females. The mean age was 69.8 ± 14.4 years, and 719 were under 65 years old. Regarding the presence or absence of disease, 1172 respondents had either physical or mental diseases. Regarding SRH, 1507 respondents were very healthy, or fairly healthy. Regarding educational backgrounds, 742 respondents had a junior college or university degree. Regarding marital status, 2206 were married, divorced, or bereaved. Regarding employment status, 1198 respondents were unemployed. Regarding annual household income, 1042 respondents had an annual household income of 3.01 million yen or more.

### 3.2 Summary of JPCAT‐SF score (Table 2)

**TABLE 2:** Breakdown of JPCAT‐SF responses, *N* = 2520.

Measurement item	Definitely yes	Probably yes	Undecided	Probably not	Not at all	Mean score
First contact: B1: “If you feel sick on a day when the doctor's office is closed, can you be seen at that doctor's office on that day?”	181 (7.1%)	664 (26.3%)	622 (24.6%)	737 (29.2%)	247 (9.8%)	46.6 ± 26.3 (0–100)
First contact: B2: “If you feel sick outside of medical hours, can you be seen at that doctor's office on that night?”	155 (6.1%)	609 (24.1%)	662 (26.2%)	751 (29.8%)	291 (11.5%)	
Continuity: C1: “Does the doctor understand you well as a person, not only as a patient with symptoms or disease?”	425 (16.8%)	781 (30.9%)	880 (34.9%)	283 (11.2%)	101 (4.0%)	62.2 ± 24.9 (0–100)
Continuity: C2: “Does the doctor know what the most important issues are for you?”	554 (21.9%)	701 (27.8%)	795 (31.5%)	254 (10.0%)	145 (5.7%)	
	Yes	No				
Coordination: D1: “Have you ever visited a specialist?”	1694 (67.2%)	715 (28.3%)				62.5 ± 25.2 (0–100)
Coordination: D2: “Did the doctor recommend you visit a specialist?”	679 (26.9%)	324 (12.8%)	391 (15.5%)	66 (2.6%)	150 (5.9%)	
Coordination: D3: “Did the doctor discuss with you the options regarding referring medical institutions?”	480 (19.0%)	447 (17.7%)	398 (15.7%)	130 (5.1%)	144 (5.7%)	
Comprehensiveness (services available when needed): E1: Whether they can consult with their doctors about “abuse”	241 (9.5%)	613 (24.3%)	831 (37.4%)	296 (11.7%)	263 (10.4%)	54.6 ± 25.3 (0–100)
Comprehensiveness (services available when needed): E2: Whether they can consult with their doctors about “how to end one's own life”	276 (10.9%)	693 (27.5%)	934 (37.0%)	234 (9.2%)	199 (7.8%)	
Comprehensiveness (services actually received): F1: Whether they had ever received advice from their doctors on “over‐the‐counter drugs and supplements”	367 (14.5%)	332 (13.1%)	334 (13.2%)	402 (15.9%)	979 (38.8%)	31.3 ± 30.1 (0–100)
Comprehensiveness (services actually received): Whether they had ever received advice from their doctors on “health information provided by media such as TV and newspapers”	186 (7.3%)	225 (8.9%)	355 (14.0%)	435 (17.2%)	1231 (48.8%)	
Community orientation: G1: “Does the doctor investigate whether the medical care meets the residents' needs?”	330 13.0 (%)	599 23.7 (%)	877 39.5 (%)	372 (14.7%)	146 (5.7%)	55.1 ± 24.3 (0–100)
Community orientation: G2: “Does the doctor conduct research on local health issues?”	253 (10.0%)	549 (21.7%)	942 (37.3%)	430 (17.0%)	132 (5.2%)	
The total score of the JPCAT‐SF						51.8 ± 16.8 (0–100)

Abbreviation: JPCAT‐SF, Japanese Primary Care Assessment Tool Short Form.

The following is an excerpt from the responses obtained at JPCAT‐SF used in the analysis. The mean score of the coordination was 62.5 ± 25.2 points. The mean score for comprehensiveness (services available) was 54.6 ± 25.3 points. The mean score of community orientation was 55.1 ± 24.3. The total score of the JPCAT‐SF was 51.8 ± 16.8 points.

### 3.3 Univariate analysis of the association of self‐rated health with the JPCAT‐SF scores and each health‐related indicator (Table 3)

**TABLE 3:** Univariate analysis of the association of self‐rated health with the JPCAT‐SF scores and each health‐related indicator (the chi‐square test).

High self‐rated health	Odds ratio	95%CI		*p* Value
*Whole group*				
Above average JPCAT‐SF score	0.90	0.75	1.06	0.21
Male	0.82	0.7	0.97	0.02
Non‐elderly	1.69	1.4	2.04	<0.001
High educational background	1.78	1.47	2.15	<0.001
Married	1.18	0.98	1.42	0.06
Not working	0.45	0.38	0.53	<0.001
Annual income >3 million yen	1.65	1.38	1.97	<0.001
*Subgroup with chronic disease*				
Above average JPCAT‐SF score	1.19	0.92	1.55	0.15
Male	0.81	0.63	1.03	0.08
Non‐elderly	1.37	1.02	1.83	0.02
High educational background	1.87	1.42	2.45	<0.001
Married	1.24	0.94	1.63	0.11
Not working	0.50	0.39	0.64	<0.001
Annual income >3 million yen	1.60	1.24	2.06	<0.001
*Subgroup without chronic disease*				
Above average JPCAT‐SF score	0.94	0.69	1.28	0.71
Male	1.43	1.04	1.98	0.02
Non‐elderly	1.19	0.86	1.66	0.25
High educational background	1.59	1.13	2.27	0.006
Married	1.29	0.92	1.80	0.11
Not working	0.61	0.45	0.83	0.001
Annual income >3 million yen	1.35	0.98	1.99	0.055

*Note*: Each variable was categorized as follows: Whether the sex is male was used as categorical data. Age was used in the analysis as “non‐elderly” if the age was less than 65. Educational background was used in the analysis as “high educational background” if the respondent had a junior college or university degree or higher. Marital status was used in the analysis of “whether the respondent has been married or not” as a confounding factor. Working status was defined in the analysis as “not working” if the respondent was unemployed or retired. Respondents were asked to answer on a 4‐point scale their annual household income (1.5, 3, and 5 million yen), and whether the income was 3.01 million yen or more in the middle was used for the analysis.

Abbreviations: CI, confidence interval; JPCAT‐SF, Japanese Primary Care Assessment Tool Short Form.

The results of the univariate analysis are presented in Table 3. Good SRH showed a significant positive correlation with “non‐elderly” (odds ratio [OR] 1.69, 95% confidence interval [CI] 1.4–2.04), “high educational background” (OR 1.78, 95%CI 1.47–2.15), annual income >3 million yen (OR 1.65, 95%CI 1.38–1.97). In contrast, good SRH showed a significant negative correlation with “not working” (OR 0.45, 95%CI 0.38–0.53).

### 3.4 Multivariate analysis of the association between good SRH and high JPCAT‐SF scores, classified with and without chronic disease (Table 4)

**TABLE 4:** Multivariate analysis of the association between JPCAT‐SF scores and self‐rated health, classified with and without chronic disease (logistic analysis).

	Odds ratio	95%CI		*p* Value
*Whole group*				
Above average JPCAT‐SF score	1.04	0.90	1.31	0.36
Male	0.89	0.73	1.07	0.24
Non‐elderly	0.97	0.76	1.24	0.83
High educational background	1.35	1.09	1.67	0.004
Married	0.97	0.78	1.19	0.79
Not working	0.51	0.41	0.63	<0.001
Annual income >3 million yen	1.25	1.02	1.53	0.028
*Subgroup with chronic disease*				
Above average JPCAT‐SF score	1.32	1.009	1.73	0.043
Male	0.83	0.63	1.09	0.19
Non‐elderly	0.79	0.55	1.13	0.20
High educational background	1.47	1.08	1.99	0.012
Married	1.02	0.75	1.41	0.86
Not working	0.55	0.40	0.75	<0.001
Annual income >3 million yen	1.32	0.99	1.75	0.051
*Subgroup without chronic disease*				
Above average JPCAT‐SF score	0.98	0.70	1.38	0.93
Male	1.44	1.02	2.05	0.037
Non‐elderly	0.81	0.52	1.26	0.36
High educational background	1.39	0.95	2.04	0.086
Married	1.09	0.76	1.58	0.61
Not working	0.58	0.38	0.88	0.012
Annual income >3 million yen	1.07	0.74	1.56	0.68

*Note*: Adjusted for sex, age, education, marriage, employment, and income factors for each group. Each variable was categorized as follows: Male sex was used as categorical data. Age was used in the analysis as “non‐elderly” if the age was less than 65. Educational background was used in the analysis as “high educational background” if the respondent had a junior college or university degree or higher. Marital status was used in the analysis of “whether the respondent has been married or not” as a confounding factor. In the analysis, working status was defined as “not working” if the respondent was unemployed or retired. Respondents were asked to answer on a 4‐point scale their annual household income (1.5, 3, and 5 million yen), and whether the income was 3.01 million yen or more was used for the analysis. The above average JPCAT‐SF score used as an explanatory variable.

Abbreviations: CI, confidence interval; JPCAT‐SF, Japanese Primary Care Assessment Tool Short Form.

In the entire JPCAT‐SF participant population and in the groups with and without chronic disease, we conducted a multivariate analysis adjusting for sex, age, education, current marital status, employment status, and annual household income as confounding factors. Good SHR and high JPCAT‐SF scores showed a significant positive correlation (OR 1.32, 95%CI 1.009–1.73) in the group with disease (Appendix S3), and no significant correlation (OR 0.98, 95%CI 0.70–1.38) in the group without disease.

### 3.5 Association between good SRH and each component of JPCAT‐SF (Tables 5 and 6)

**TABLE 5:** Each JPCAT‐SF components associated with good SRH (the chi‐square test).

	Odds ratio	95%CI		*p* Value
*Whole group*				
First contact	0.96	0.81	1.13	0.62
Longitudinality	1.00	0.85	1.18	0.96
Coordination	0.71	0.60	0.83	<0.001
Comprehensiveness available	1.18	1.007	1.40	0.03
Comprehensiveness provided	0.92	0.76	1.11	0.40
Community orientation	1.08	0.92	1.27	0.32
*Subgroup with chronic disease*				
First contact	1.13	0.88	1.45	0.30
Longitudinality	1.18	0.93	1.51	0.15
Coordination	0.89	0.70	1.13	0.34
Comprehensiveness available	1.35	1.06	1.72	0.01
Comprehensiveness provided	1.22	0.93	1.60	0.12
Community orientation	1.13	0.89	1.44	0.28
*Subgroup without chronic disease*				
First contact	0.91	0.66	1.23	0.53
Longitudinality	1.42	1.03	1.97	0.02
Coordination	0.81	0.60	1.11	0.18
Comprehensiveness available	1.34	0.99	1.83	0.047
Comprehensiveness provided	0.82	0.58	1.17	0.25
Community orientation	0.96	0.70	1.30	0.79

*Note*: Each component was analyzed as whether the average was above the mean score.

**TABLE 6:** Each JPCAT‐SF components associated with good SRH (logistic analysis).

	Odds ratio	95%CI		*p* Value
*Whole group*				
First contact	1.08	0.90	1.30	0.35
Longitudinality	1.11	0.92	1.33	0.25
Coordination	0.75	0.62	0.90	0.002
Comprehensiveness available	1.30	1.09	1.56	0.004
Comprehensiveness provided	0.86	0.70	1.06	0.16
Community orientation	1.26	1.05	1.51	0.01
*Subgroup with chronic disease*				
First contact	1.25	0.96	1.63	0.08
Longitudinality	1.24	0.96	1.61	0.09
Coordination	0.88	0.68	1.13	0.33
Comprehensiveness available	1.44	1.11	1.87	0.005
Comprehensiveness provided	1.18	0.88	1.57	0.24
Community orientation	1.25	0.96	1.62	0.09
*Subgroup without chronic disease*				
First contact	1.03	0.74	1.44	0.82
Longitudinality	1.49	1.04	2.12	0.02
Coordination	0.74	0.53	1.05	0.09
Comprehensiveness available	1.46	1.04	2.03	0.02
Comprehensiveness provided	0.70	0.48	1.02	0.07
Community orientation	1.11	0.79	1.54	0.53

*Note*: Each component was analyzed as whether the average was above the mean score. Adjusted for sex, age, education, marriage, employment, and income factors for each group. Each variable was categorized as follows: Male sex was used as chategorical data. Age was used in the analysis as “non‐elderly” if the age was less than 65 years old. Educational background was used in the analysis as “high educational background” if the respondent had a junior college or university degree or higher. Marital status was used in the analysis of “whether the respondent having be married or not” as a confounding factor. In the analysis, working status was defined as “not working” if the respondent was unemployed or retired. Respondents were asked to answer on a 4‐point scale their annual household income (1.5, 3, and 5 million yen), and whether the income was 3.01 million yen or more was used for the analysis. The above average JPCAT‐SF score used as an explanatory variable.

Abbreviations: CI, confidence interval; JPCAT‐SF, Japanese Primary Care Assessment Tool Short Form.

Good SRH was positively associated with comprehensiveness available (OR 1.30, 95%CI 1.09–1.56) and community orientation (OR 1.26, 95%CI 1.05–1.51). In contrast, good SRH was negatively associated with coordination (OR 0.75, 95%CI 0.62–0.9).

## 4 DISCUSSION

In Japan, our study revealed that good SRH among rural residents with chronic diseases was associated with quality of primary care. Previous studies conducted in Korea,^9^ China,^10^ and rural Malawi^17^ found positive correlations between high SRH and PCAT scores. The target populations of the previous studies were all conducted in primary care facilities, which is consistent with the present study. SRH predicted 5‐year mortality^7^ and living conditions.^8^ Thus, high‐quality primary care might contribute to these health outcomes by improving SRH. In addition, our study was conducted on the general population as a whole, whereas previous studies interviewed patients who visited a medical institution. To the best of our knowledge, no similar studies have been conducted in the entire community, and our study is unique in that a broad survey was conducted including those who visited a family practice outside the research institution's facilities. In a society with a declining birthrate and aging population, many services have been forced to downsize. Our results highlighted the importance of maintaining high‐quality primary care in rural areas.

The resources for long‐term care services in the surrounding areas were limited, and it was challenging to receive medical and long‐term care services in familiar areas with peace of mind. Older people also have high rates of multimorbidity.^18^ A study on primary care at a Community Health Center in Shanghai, China^19^ showed that multimorbidity (having two or more diseases) and SRH were positively correlated with PCAT scores. The target population of our study in the group with chronic diseases included multimorbidity patients. This result supported the fact that JPCAT scores were higher in the group with chronic diseases.

Here, we discuss the association between good SRH and each component of the JPCAT‐SF. A positive association is observed between comprehensiveness available and community orientation. The questions about comprehensiveness available included consultations with doctors regarding how to end one's own life. This highlights the importance of advance care planning (ACP). The discussion of death is often avoided in clinical practice. However, ACP does not negatively affect the hopes of patients with advanced cancer, even slightly reducing their anxiety.^20^ In a super‐aged society like Japan,^2^ ACP might be useful for residents' health during primary care practice. The questions about community orientation included the understanding of the medical needs and local health issues of the community through investigation and research. A report suggests that to capture and monitor the whole picture of modern community medicine in Japan, the key viewpoints are “community,” “care systematization,” and “coworking with residents”.^21^ Coworking with residents to create a medical system that fits their local context may influence their health. The negative association with coordination was easy to understand. In most cases where patients are referred, they have low SRH because they are seriously ill or require advanced treatment.

In our study, no correlation was found between high JPCAT‐SF scores and good SRH in the group of persons without chronic diseases who had a family doctor. This might be due to the fact that the medical treatment for health problems was solved in a short period of time and continuous visits were unnecessary, which might not have a significant impact on the health views and concerns of the respondents.

When looking at the association between the presence or absence of chronic disease and SRH, one report showed no significant association between pain, disability, and SRH views in the group without chronic disease,^22^ which supports the above reason.

In addition, the stratified analysis of SRH in our study showed that the group with no chronic diseases had a higher SRH with almost 4–5 points (Table 1). Therefore, one of the factors that showed no association in our study may be that the respondents were confident about their health and did not feel any difference in their responses to their primary care provider.

### 4.1 Limitations

First, this was a cross‐sectional study. We could not show a causal relationship between the JPCAT‐SF and SRH. Therefore, a longitudinal study that includes other rural areas is needed. Secondly, this was a single‐region study. Hence, these results may differ in other rural settings such as remote islands. Third, the populations included had participants with a high interest in health. Thus, their JPCAT and SRH scores may have been higher than those of the actual population.

## 5 CONCLUSION

Favorable patient experiences in the primary care field were associated with good SRH among Japanese residents with chronic diseases in rural areas.

## CONFLICT OF INTEREST STATEMENT

The author (Hisata Y) received the 2021 JA Mutual Aid Federation commissioned research project and the 34th regional health care research grant for the KYANS study.

## ETHICS APPROVAL STATEMENT

This study was approved by the Ethics Committee of Nagahama City Kohoku Hospital (Approval No. 3 in FY 2019, No. 1 in FY 2021, and No. 1 in FY 2023).

## PATIENT CONSENT STATEMENT

We obtained consent after clearly stating that respondents were free to respond or withdraw their responses.

## CLINICAL TRIAL REGISTRATION

None.

## Supporting information


Appendix S1:

## Data Availability

The data that support the findings of this study are available from the corresponding author, Yoshio Hisata, upon reasonable request.
